# Comparative Genomic Analyses and CRISPR-Cas Characterization of *Cutibacterium acnes* Provide Insights Into Genetic Diversity and Typing Applications

**DOI:** 10.3389/fmicb.2021.758749

**Published:** 2021-11-03

**Authors:** Natalia Cobian, Allison Garlet, Claudio Hidalgo-Cantabrana, Rodolphe Barrangou

**Affiliations:** ^1^Department of Food, Bioprocessing and Nutrition Sciences, North Carolina State University, Raleigh, NC, United States; ^2^BASF Corporation, Tarrytown, NY, United States

**Keywords:** CRISPR, genomics, Cas, genotyping, phylogeny

## Abstract

*Cutibacterium acnes* is an important member of the human skin microbiome and plays a critical role in skin health and disease. *C. acnes* encompasses different phylotypes that have been found to be associated with different skin phenotypes, suggesting a genetic basis for their impact on skin health. Here, we present a comprehensive comparative analysis of 255 *C. acnes* genomes to provide insights into the species genetic diversity and identify unique features that define various phylotypes. Results revealed a relatively small and open pan genome (6,240 genes) with a large core genome (1,194 genes), and three distinct phylogenetic clades, with multiple robust sub-clades. Furthermore, we identified several unique gene families driving differences between distinct *C. acnes* clades. Carbohydrate transporters, stress response mechanisms and potential virulence factors, potentially involved in competitive growth and host colonization, were detected in type I strains, which are presumably responsible for acne. Diverse type I-E CRISPR-Cas systems and prophage sequences were detected in select clades, providing insights into strain divergence and adaptive differentiation. Collectively, these results enable to elucidate the fundamental differences among *C. acnes* phylotypes, characterize genetic elements that potentially contribute to type I-associated dominance and disease, and other key factors that drive the differentiation among clades and sub-clades. These results enable the use of comparative genomics analyses as a robust method to differentiate among the *C. acnes* genotypes present in the skin microbiome, opening new avenues for the development of biotherapeutics to manipulate the skin microbiota.

## Introduction

The Gram-positive anaerobic *Cutibacterium* (formerly *Propionibacterium*) *acnes* is one of the most dominant species in the pilosebaceous follicle and a key member of the human skin microbiome (Dreno et al., 2018). Originally classified and named as *Propionibacterium acnes*, it was recently reclassified as *Cutibacterium acnes* (Scholz and Kilian, 2016), a nomenclature broadly adopted despite discordance in the scientific community (Alexeyev et al., 2018; Dekio et al., 2019). Recent skin microbiome studies have attempted to decipher differences in microbial populations associated with healthy skin vs. disease states, and potential associations with gut microbiome composition and host immune health (Lee et al., 2019). Several studies have established that *Cutibacterium*, *Corynebacterium*, *Staphylococcus*, and *Streptococcus* are important members of the skin microbiota (Christensen et al., 2016; Barnard and Li, 2017) and observed a reduction in *Cutibacterium* relative abundance in certain skin diseases such as psoriasis, atopic dermatitis and rosacea (Kong et al., 2012; Chang et al., 2018; Woo et al., 2020). Due to the clinical importance of acne vulgaris, there is increasing interest in studying the skin microbiome and deciphering differences between healthy and disease populations regarding microbial, immunological and hormonal factors (Lee et al., 2019; McLaughlin et al., 2019; Ramasamy et al., 2019; Szegedi et al., 2019), as well as in other skin disease like rosacea (Thompson et al., 2020; Woo et al., 2020).

*Cutibacterium acnes* has several distinct genotypes within this species which can co-exist in healthy skin but lead to acne in cases where relative abundance shifts (McLaughlin et al., 2019). Three phylogroups or clades have been described, type I, II and III (McDowell et al., 2008), with several sub-clades described within type I (McLaughlin et al., 2019). While the type I clade is widely present in healthy individuals it is also enriched in acne vulgaris, with significantly higher relative abundance of sub-clades IA_1_ and IC. In contrast, sub-clades IA_2_, IB, and type II are more prevalent on healthy skin (Fitz-Gibbon et al., 2013; McLaughlin et al., 2019). Noteworthy, while type I and type II clades are present in both healthy and acne skin, albeit in different proportions. The type III clade is exclusively present in healthy skin and absent from acne-associated samples (McLaughlin et al., 2019), though it can be present in other skin diseases such as progressive macular hypomelanosis (Barnard et al., 2016; Petersen et al., 2017). Overall, a healthy skin typically presents a more balanced distribution of diverse *C. acnes* phylotypes compared to acnes disease, where lower diversity is often associated with type IA_1_ enrichment. As a result of the genetic diversity of *Cutibacterium*, recent efforts have focused on taxonomy and genotyping to compare strains associated with acne vulgaris. Originally, methods were based on PCR amplification and sequencing of variable sequences such as 16S rRNA, *RecA*, and *tly*, as well as multilocus sequence typing (MLST) encompassing seven to nine genes (McDowell et al., 2011, 2012, 2013; Dreno et al., 2018; Bangayan et al., 2020). More recently, the advent of next-generation sequencing (NGS) and bioinformatic technologies has enabled the determination of the genomes of numerous isolates. However, no extensive comparative genomic analysis has been performed to date in *C. acnes*, nor have their CRISPR-Cas systems been characterized and used for genotyping purposes. Unraveling the unique genetic determinants associated with each clade and sub-clade of this particular species will provide new insights into the skin microbiome, the virulence or pathogenicity of specific sub-clades and the skin virome based on the CRISPR spacers content.

Here, we carried out a comparative genomic analyses of *C. acnes* to investigate the phylogenetic relationship among 255 *C. acnes* strains, characterized CRISPR-Cas loci and provide insights into key genetic determinants that account for specific clades and sub-clades.

## Materials and Methods

### Genome Annotation

The 255 chromosomal sequences of *Cutibacterium acnes* available at NCBI on October 2019 were retrieved (Supplementary File 1). To ensure consistency in open reading frame (ORFs) identification and annotation, all genomes were reannotated with Prokka v1.13.3 with standard options (Seemann, 2014), using Prodigal (Hyatt et al., 2010) for gene prediction.

### Core and Pan Genome Analyses

The core and pan genome analyses were carried out using Roary v3.12.0 (Page et al., 2015) with the flags -env -i 95 -cd 100, for a minimum percentage identity of 95% for BLASTp and strict threshold for the consideration of core genes, only if they occur in 100% of the genomes. Briefly, the predicted ORFs of each genome were used to perform the pan genome analyses to identify the total genes present in the pan genome, core genes, unique genes and new genes. The pan genome and new genes graph were depicted in RStudio v1.1.463 (R Core Team, 2015) using the create_pan_genome_plots script^1^. The openness of the pan genome was calculated according to Heap’s law (Tettelin et al., 2008). The functional COGs were assigned to pan-, core- and unique genes using EggNOG 5.0^2^ (Huerta-Cepas et al., 2019) and then depicted in RStudio using ggplot2 (Wickham, 2009).

The heatmap containing the gene presence/absence results of the pangenome was generated using roary_plots.py script^3^. A customized version was generated in RStudio using heatmap.2^4^ performing hierarchical clustering for both, genomes (rows) and genes (columns). The unique genes existing in each clade were identified using the query_pan_genome option implemented in Roary v3.12.0 (Page et al., 2015) with the flags: -a difference -g clustered_proteins -c 100. Similarly, the common genes shared among two groups were identified using the query_pan_genome with the flags: -a intersection -g clustered_proteins -c 100. The Venn Diagram was depicted in RStudio using the VennDiagram package.

### Phylogenomic Analyses

The 1,194 core genes shared among the 255 *C. acnes* strains were aligned using the PRANK algorithm implemented in Roary v3.12.0 (Page et al., 2015) while performing core- pan genome analysis (see previous section). The resulting multi-FASTA alignment file was used as input in FastTree^5^ to infer the phylogenomic tree with generalized time reversible model (GTR), using standard flags -nt -gtr. The resulting output newick format file was used to depict the final tree using FigTree v1.4.4^6^.

### Identification of Virulence Factors

Virulence factors within *C. acnes* genomes were investigated using BLAST +. Briefly, a local blastx was performed creating a database with the amino acid sequence of previously identified virulent factors on *C. acnes* KPA171202 (McLaughlin et al., 2019; Supplementary Table 3), a type IB strain, to search among the other 254 genomes used as query. The results were hand-curated and only proteins with 70% identity across the full alignment were considered further. Finally, the blastx results for each virulent protein were represented as a heatmap displaying the percentage of identity with a blue gradient using the Heatmap package^7^ in RStudio (R Core Team, 2015), also representing the absence of each protein in white when applicable.

### CRISPR-Cas System Identification and Characterization

CRISPR-Cas systems were identified and subtyped using CRISPRdisco providing the .fna and .faa files of each of the 255 *C. acnes* annotated genomes as input (Crawley et al., 2018). After identification and manual curation of each CRISPR-Cas locus, the downstream and upstream regions were analyzed to identify the overall chromosomal location and nucleotide conservation of *cas* genes and flanking sequences. Then, specific strains were selected as representatives for each CRISPR subtype to manually depict CRISPR loci of interest.

The Cas proteins alignment among *C. acnes* strains and with *E. coli* K12 type I-E was performed using ClustalW to identify the amino acid identity similarity. Then, the concatenated Cas proteins tree was generated using the genetic distance model Jukes-Cantor and the Neighbor-Joining tree build method. The final tree was depicted using FigTree v1.4.4 (see text footnote 6).

The CRISPR array, including the CRISPR repeats and CRISPR spacers, were identified, automatically extracted, visualized and aligned using CRISPRviz (Nethery and Barrangou, 2019a). Then, spacers for each strain were concatenated using the append_spacers.sh script implemented in CRISPRutils (Nethery and Barrangou, 2019b)^8^. The target (protospacer) identity from putative invasive nucleic acids was analyzed using BLAST (Camacho et al., 2009), with the flags: blastn -evalue 1e-3 -remote -db nt -outfmt 5. The results were trimmed to discard matches not related to phage, plasmid or prophages using blast_parser.py with the flags -ct implemented in CRISPRutils. The trimmed output file was used to depict a heatmap in RStudio, reflecting the number of positive hits in different targets. Finally, the alignment of the spacer-protospacer match also containing the flanking regions was extracted using blast_parser.py with the flag -p as implemented in CRISPRutils. The flank alignment file was used to represent the protospacer adjacent motif (PAM) sequence in WebLogo server (Crooks et al., 2004) based on a frequency chart where the height of each nucleotide represents the conservation of that nucleotide at each position.

The CRISPR repeat sequence was subjected to RNA folding analyses using NUPACK^9^ (Zadeh et al., 2011) to predict the hairpin structure of the mature CRISPR RNA (crRNA) and depicted by hand.

### Statistical Analyses

Data distribution was analyzed using the Shapiro–Wilk test for genome size, %GC content, and number of predicted genes, under the hypothesis that each variable follows a Gaussian distribution. The resulting *p*-values < 0.05 displayed no normal distribution and therefore, non-parametric tests were used for subsequent analysis. The Kruskal–Wallis test was used to compare the number of genes between strain types to assess differences among groups, under the hypothesis of equal means among groups. Comparisons with a *p*-value < 0.05 were considered statistically significant. The statistical analyses were performed in R studio v1.1.463.

## Results

### *Cutibacterium acnes* Core and Pan Genome Determination

**FIGURE 1:**
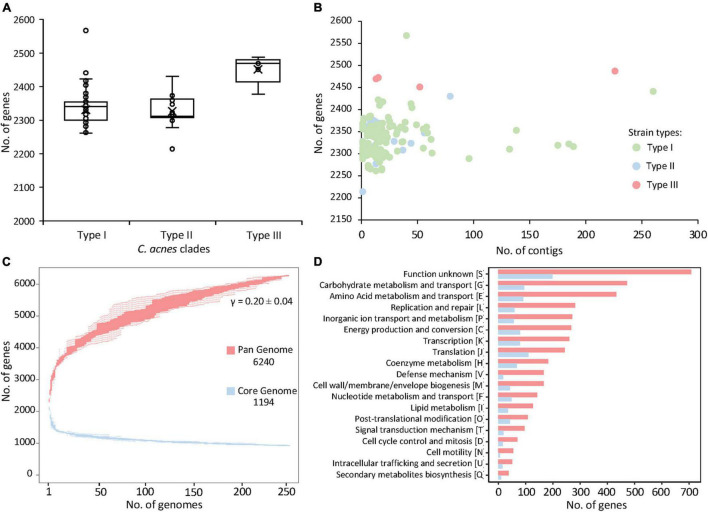
Pan and core genome of the 255 *Cutibacterium acnes*. **(A)** Distribution of the number of genes per genome with *C. acnes* strains organized by clades. **(B)** Number of genes plotted against the number of contigs of the same genome. **(C)** The number of genes increases in the pan genome (red) and decreases in the core genome (blue) with the addition of more genomes. **(D)** Number of genes in the pan genome (red) and core genome (blue) corresponding to each functional category.

The initial genome screening of the 255 genomes of *C. acnes* publicly available at NCBI (October 2019) (Supplementary File 1) did not reveal significant overall differences in genome size (2.51 Mbp ± 0.04) (Mean ± SD), or GC content (60.1% ± 0.08). The number of putative protein-coding sequences in genomes varied between 2,214 and 2,567, with an average of 2,331 ± 40.31. However, there was a significantly (*p*-value < 0.005) higher number of genes detected in type III strains (2,469 ± 38.82) (Figure 1A). Type III strain had a larger genome size (65 Kb) than the average type I and type II strains (2.57 Mbp ± 0.04 vs. 2.51 Mbp ± 0.036, respectively), although differences in available genomes for each strain type (203 type I, 47 type II, 5 type III) may have influenced this observation. No correlation was observed between the number of genes predicted and the number of contigs of the genome, suggesting that sequencing depth and genome assembly quality was not an influencing factor for subsequent analyses (Figure 1B). In fact, 80.78% (206/255) of the genomes have less than 20 contigs. Overall, the “typical” *C. acnes* genome is similar to *C. avidum* (2.5 Mbp), but slightly bigger than other *Cutibacterium* species like *C. granulosum* (2.18 Mbp). Noteworthy, the *Propionibacterium* genus, where *C. acnes* was previously classified (Scholz and Kilian, 2016), contains species which genome size ranging between 2.6 and 5 Mbp, with a 67% GC content, relatively higher than the 60% displayed by *C. acnes*.

To assess genomic conservation across the 255 strains, the total coding sequences were used to determine the pan genome using Roary (Page et al., 2015). This analysis revealed a total of 6,240 genes representing the pan genome of 255 *C. acnes* strains (Figure 1C). Remarkably, the pan genome did not reach a plateau, as the addition of each new genome still increased the number of genes in the pan genome (Figure 1C). The Heap’s law calculation (Tettelin et al., 2008) corroborated the openness (γ > 0) of this pan genome with γ = 0.20 ± 0.04 and *K* = 3.32 ± 0.10. Of the 6,240 identified coding sequences, 1,194 represent the core genes, shared across all strains. There are 5,046 accessory genes that are present in the pan genome outside the core genes, which contributes to major genetic and presumably phenotypic differences among strains.

The functional categories for the core and pan genes were annotated through EggNOG (Huerta-Cepas et al., 2019) and the number of genes for each functional category was represented with bar graphs (Figure 1D). The majority of the core genes were related to basic biological functions such as carbohydrate and amino acid metabolism, energy production, transcription and translation, and coenzyme metabolism; besides the genes of unknown function. Similar trends were observed with the predicted functional categories of the coding sequences of the pan genome (Figure 1D).

**FIGURE 2:**
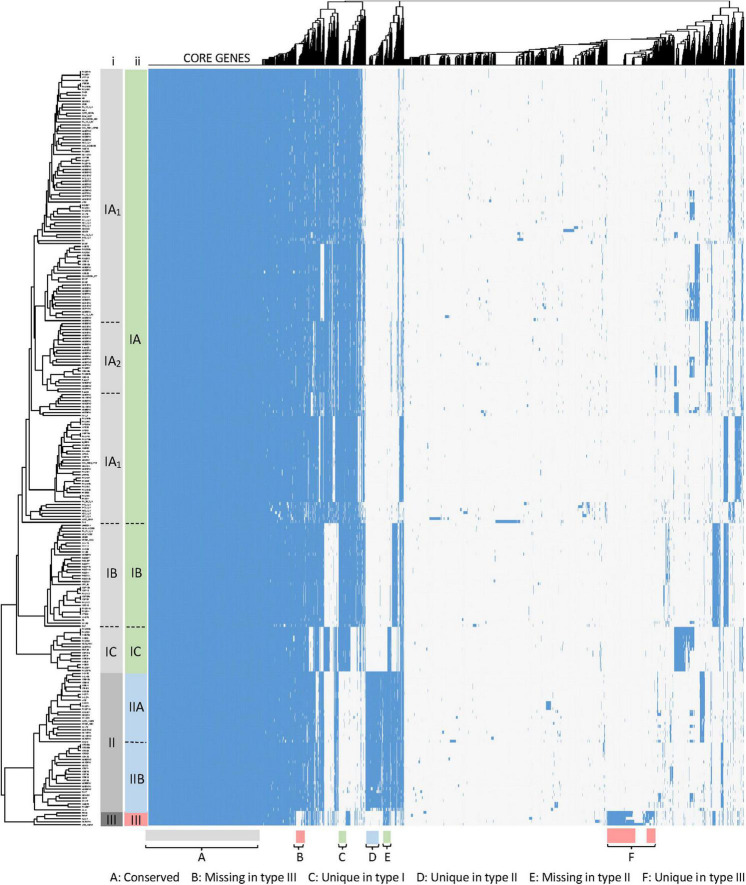
Gene cluster matrix of presence/absence (blue)/(white) of the 6,240 genes (columns) that constitute the pan genome of the 255 *C. acnes* strains (rows). Hierarchical clustering was performed for both rows and columns and dendrograms were depicted. The main clades and sub-clades of strains are labeled (i) in gray according to the current nomenclature and (ii) color coded for type I, type II and type III, according to our newly proposed classification (see Figure 3). Signature gene blocks of each clade are indicated in the bottom.

**FIGURE 3:**
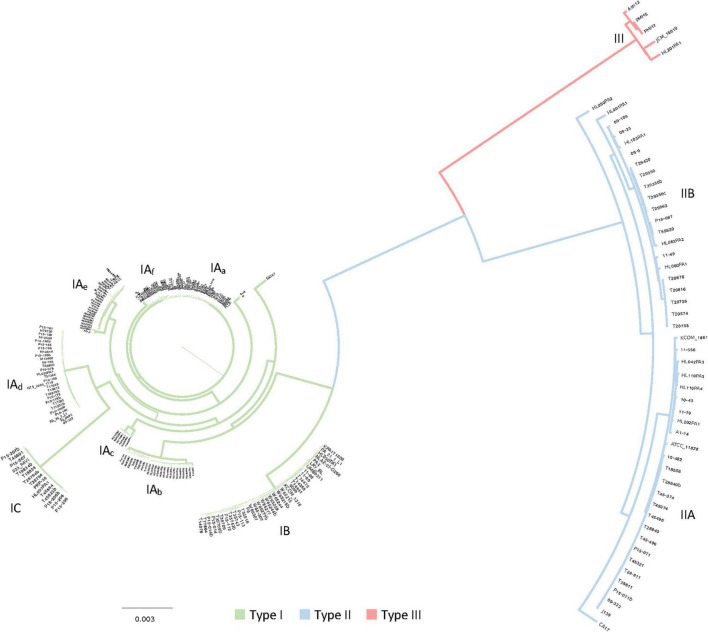
Core genome based phylogenomic tree with the 1,194 core genes detected in the 255 *C. acnes* strains. The main clades of strains were identified, and color coded in the branches for type I, type II and type III, with green, blue and red respectively. Sub-clades within the type I (IA, IB, and IC) and type II clades (IIA, IIB) were also identified, together with six sub-groups within the sub-clade IA, labeled as IA_a_ to IA_f_.

The overall visualization of 6,240 genes occurrence across 255 genomes (1.6 M data points) using a heatmap revealed several distinct groups and subgroups of strains based on the gene presence/absence (Figure 2). For instance, the dendrogram of the hierarchical clustering displayed that two major clades exist, with the bottom branch divided in two sub-clades. These three clades correspond to the three phylogenetic groups previously described in *C. acnes* and named as type I, II and III strains clades (McLaughlin et al., 2019). The major type I clade (green), containing the majority of genomes, is distinct and separate from *C. acnes* type II clade (blue) and *C. acnes* type III clade (red). Noteworthy, the type I clade is further segregated in several sub-clades, that correlate with some of the previously described sub-clades IA, IB and IC (Lomholt and Kilian, 2010; Bruggemann et al., 2012; McLaughlin et al., 2019). The current nomenclature distinguishes only two sub-groups within the sub-clade IA (IA_1_ and IA_2_) (Figure 2, column i), however, no consistency in the IA_1_-IA_2_ sub-grouping was identified in our analysis (Figure 2, column ii), where several sub-groups exist (see Figure 3). Regarding the type II clade, we identified two main sub-clades (IIA and IIB), that were not previously established. Overall, these results confirm the presence of three main types and several sub-types within the type I and type II clades.

Besides the strain clustering, it is important to visualize the gene occurrence patterns (Figure 2), revealing the relative size of the core genome, and variability of the genes representing the pan genome, with groups of genes uniquely present/absent in the three clades (Figure 2, bottom part), and clusters of genes accounting for the various type I subtypes and type II subtypes.

### Phylogenomics of *Cutibacterium acnes*

To further decipher the phylogenetic relationship among the strains we generated a phylogenomic tree based on the alignment of the 1,194 core genes previously detected (see section above). The results of this analysis are consistent with the heatmap of the pan genome, displaying a clear segregation of the three major clades, and the presence of multiple type I sub-clades and type II sub-clades (Figure 3). Within the sub-clade IA, six different subgroups were identified, potentially corresponding to six different genotypes, labeled as IA_a_ to IA_f_ according to the clustering reflected in this phylogenomic tree based on 1,194 core genes (Figure 3). Likewise, the newly proposed sub-clades IIA and IIB, identified in the pan genome results (Figure 2), were also identified here in the phylogenomic tree using the core genes, highlighting the consistency of the strain and genome clustering results. The overall distribution of the 255 genomes among clades and sub-clades, after the phylogenomic analyses was as follow: 153 type IA, 35 type IB, 15 type IC, 24 type IIA, 23 type IIB and 5 type III.

### Unique Genes in Various *Cutibacterium acnes* Clades

**FIGURE 4:**
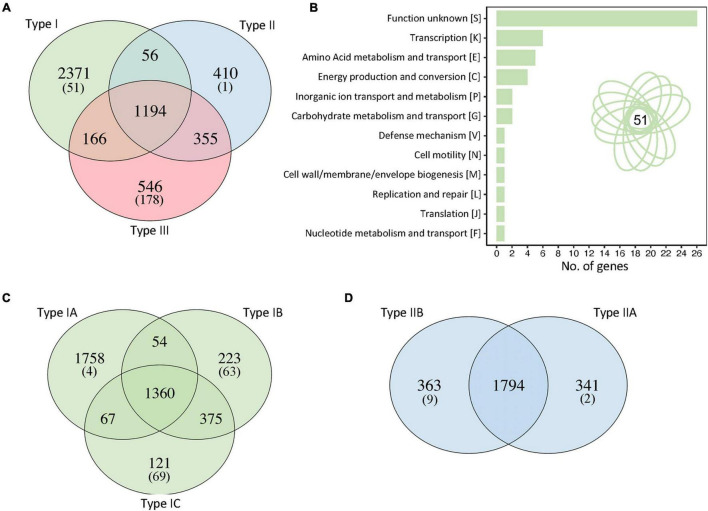
Unique genes in each *C. acnes* clade. **(A)** Identification of the number of unique genes for each clade, genes shared by two clades and core genes. The number between brackets represent the number of unique genes present in all the strains of that particular clade. **(B)** Functional categories of the 51 unique genes present in all type I strains. Manual curation and annotation of these 51 unique genes allowed to elucidate their functionality (see Supplementary Tables 1, 2). **(C)** Unique genes and shared genes within the sub-clades of type I strains. **(D)** Unique genes and shared genes within the sub-clades of type II strains.

To investigate the genetic determinants that drive the differentiation between different strain types, we identified unique genes that occurred in each *C. acnes* clade, using the query_pan_genome option implemented in Roary (Page et al., 2015). Interestingly, 2,371 genes occurred uniquely in type I strains (in at least one strain) whereas 410 unique genes were identified in type II clade including *cas* genes from CRISPR-Cas systems (see the corresponding section), and 546 in type III (Figure 4A). These numbers drastically decrease to 51, 1, and 178 unique genes for each clade (I, II, III, respectively) when only the unique genes present in all the strains of each clade were considered. These results suggest that most of the unique genes of each clade are only present in some strains (strain genetic variability) with few of them driving the uniqueness and differentiation of that clade.

Then, we focused the downstream analyses on type I clade to understand the genetic determinants of this group related with acne vulgaris disease. From the 2,371 unique genes, only 51 are present in all the type I strains, perhaps representing the key genetic content that distinguishes type I strains from the other two clades (Figure 4B). Functional categories were analyzed and after annotation and manual curation of the 51 genes, 71% of them were assigned to specific functions, although 29% remain with unknown functions (Supplementary Table 1). Interestingly, several unique genes present in type I *C. acnes* strains are related to carbohydrate metabolism and sugar transporters, like glycosyl hydrolase, trehalose transporters *sugA*, PTS transporters and ABC transporters; and two of the unique genes displayed homology to the type II toxin-antitoxin system HicAB from *E. coli* and other pathogens (Supplementary Table 1). Other unique genes identified in type I strains are related to energy production, transport and metabolism pathways such as ATPase associated diverse cellular activities (AAA proteins), *N*-acetyltransferases, cyanate permease, metal-dependent amidase/carboxypeptidase, and few transcriptional regulators, among others (Supplementary Table 1).

To further investigate the sub-clade clustering in type I strains, the unique genes and core genes within type I sub-clades were identified (Figure 4C). The type IA sub-clade, containing the higher number of strains, also contains the higher number of unique genes (1,758) due to strain diversity, with only four of these unique genes shared among all type IA strains (Figure 4C). Interestingly, sub-clade IB and IC have a similar number of unique genes present in all strains of each sub-clade, 63 and 69 respectively, despite the number of genomes of each sub-clade (35 and 15 respectively), although the total number of unique genes are different (223 and 121). A higher number of genomes increases strain diversity genotype and therefore the number of unique genes. Noteworthy, sub-clades IB and IC shared a higher number of core genes than the other subclades (IA-IB or IA-IC), representing a close phylogenic relationship, as previously established by the pan genome clustering (Figure 2).

Regarding the type II clade, we described the existence of two clear sub-clades, IIA and IIB, based on our pan genome results and phylogenomic analyses with core genes (Figures 2, 3), that were not previously documented in the literature. The unique genes analyses displayed more than three hundred genes in each sub-clade accounting for this sub-aggrupation, but with only a handful of genes present in all the strains of each sub-group (Figure 4D). In this regard, the type IIA clade only displayed two unique genes shared across all twenty-four strains, and these two genes are *gatC2* (part of a galactitol-specific PTS system) and NAD-dependent glycerol-3-phosphate dehydrogenase (Supplementary Table 2). For subclade IIB, nine unique genes are present in every single strain out of the twenty-three genomes within of this group, with certain genes related to maltose metabolism, ABC transporter and others (Supplementary Table 2).

### Virulence Factors

We investigated the presence of thirty-three virulence factors across all studied genomes and specifically determined whether they could account for *C. acnes* clade or sub-clade differences in virulence, especially for type I strains in general, and IA strains in particular. Surprisingly, 27 out of 33 virulent proteins were detected in all of the strains, regardless of clade and sub-clade, albeit with slight amino acid sequences differences among strains (Supplementary Figure 1A). Three of the PTRs proteins (PTR-3, PTR-4, PTR-6) were missing in few strains but overall seemed widely distributed across groups. Noteworthy, the amino acid identity of these widely distributed genetic determinants account for the strain clustering in the previously described *C. acnes* clades (I, II, III) including the differentiation of sub-clades IIA-IIB.

Interestingly, the hyaluronate lyase (HYL), an enzyme involves in the degradation of the main polysaccharide component of the dermal and epidermal matrix (hyaluronan or hyaluronic acid), was detected in both type I and type II clades but completely absent in the type III clade, which correlates with its absence in acne-associated skin. More importantly, the PTR-1_PPA0180 was uniquely detected in type I strains and is very conserved across strains, with no differences in amino acid identity (Supplementary Figure 1A). On the contrary, DeoR_PPA0299 which is a repressor of porphyrin biosynthesis, was absent in some sub-clades of type I, but present in others, and also present in most of type II strains, and all type III genomes.

The Christie Atkins Munch-Petersen (CAMP) factors are membrane pore-forming toxins that act as host tissue degradation enzymes, with a hemolytic and cytotoxic effect involved in colonization and inflammation, and potentially implicated in acne development (Christie et al., 1944; Wang et al., 2018). However, no differences were detected across the five CAMP factors analyzed across strain types. Rather, these virulent proteins seem widespread among *C. acnes* and not specifically associated with any type. Slight differences in the amino acid identity of the CAMP1_ PPA1340 were detected between type I strains and the type II and type III subsets, ranging from 95.5–100%, with similar findings for CAMP2_PPA0687 (Supplementary Figure 1A).

Finally, the chromosomal location of these 33 virulent genes was investigated to assess the potential existence of genomic islands associated with pathogenicity. However, the virulent factors seem to be widespread across the genome and not located in particular islands, as illustrated using the genome of the type IB strain KPA171202 (Supplementary Figure 1B).

### CRISPR-Cas System Identification and Characterization

CRISPR-Cas systems constitute the adaptive immune system of bacteria, against invasive genetic elements such as phages and plasmids (Barrangou et al., 2007). Here, we determined the occurrence and diversity of CRISPR-Cas systems across the 255 *C. acnes* strains, using previously developed pipelines (Crawley et al., 2018).

**FIGURE 5:**
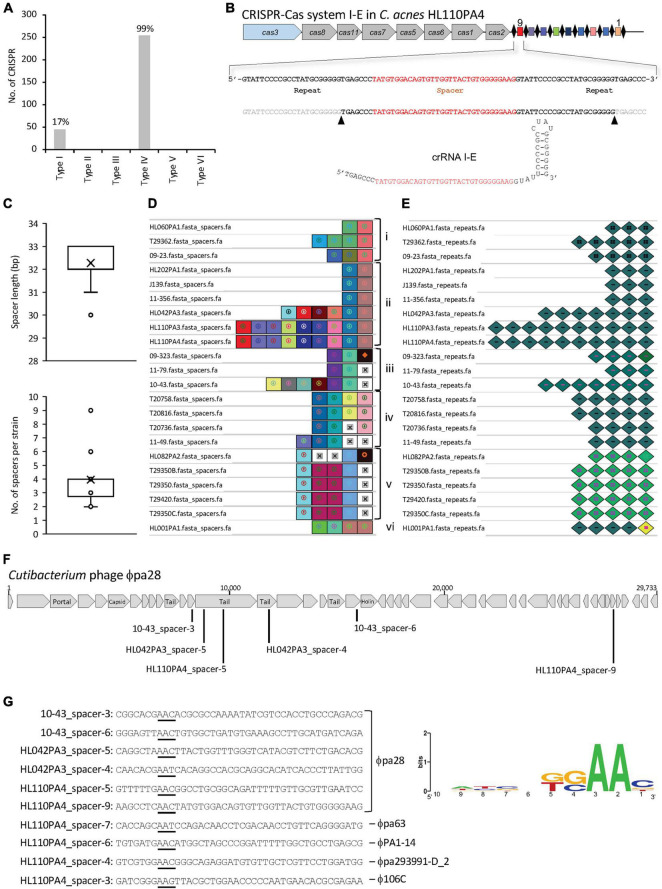
CRISPR-Cas systems in *C. acnes*. **(A)** Occurrence and diversity of CRISPR-Cas systems in 255 *C. acnes* strains. **(B)** CRISPR-Cas system I-E displaying a canonical profile with signature *cas3* gene colored in blue, Cascade in gray, CRISPR array with the repeats as black diamonds and the spacers as colored squares; with the mature CRISPR RNA (crRNA) displaying the hairpin structure on the bottom. **(C)** Distribution of the spacer length and number of spacers per strain. **(D)** Visualization of the CRISPR spacers with unique colors and shapes for each unique spacer sequence, aligned based on nucleotide identity for genotyping purposes, with the oldest spacer (ancestor) on the right and the most recently acquire spacer on the left. Groups of strains based on CRISPR spacers-genotyping are labeled from i-vi. **(E)** Visualization of the CRISPR repeats with unique colors and shapes for each unique repeat sequence. Mutated terminal repeats (corresponding to the ancestor) were identified. **(F)** Schematic representation of the *Cutibacterium* phage ϕpa28 targeted by several CRISPR spacers of various *C. acnes* strains. **(G)** Protospacer sequences (targeted by CRISPR spacers) with the corresponding protospacer adjacent motif (PAM) on the 5′-end underlined, and representation of the PAM conservation (right panel).

Overall, 45 CRISPR-Cas systems subtype I-E were identified (Figure 5A), based on the presence of the corresponding signature *cas* genes. Interestingly, these 45 type I-E CRISPR-Cas systems occurred exclusively in *C. acnes* type II strains, and not in type I nor type III strains, in concordance with previous results of a smaller dataset (Bruggemann et al., 2012). Moreover, the type I-E CRISPR-Cas systems seems to be widespread among type II strains with 95.74% strains (45/47) carrying a locus. The detailed analyses of these CRISPR-Cas loci revealed a canonical structure for this subtype I-E (Makarova et al., 2018), encompassing the signature nuclease *cas3* involved in DNA cleavage, the associated *cas* genes that constitute the Cascade complex (Cas8-Cas11-Cas7-Cas5-Cas6) (Brouns et al., 2008), and the *cas1* and *cas2* genes, involved in new spacer acquisition (Figure 5B). The sequence comparison of the Cas proteins among the 45 *C. acnes* strains displayed high identity (99%) among strains, whereas the amino acid sequence and protein length was distant from the canonical *E. coli* I-E Cas proteins with less than 30% identity (Supplementary Figure 2).

Then, the CRISPR array, encompassing the repeat and spacer sequences, was identified for 22 of the 45 CRISPR-Cas systems previously mentioned, with the remaining 23 not encoding a CRISPR array. For these 22 strains, high *cas* sequence conservation was detected across strains (99.25% ± 0.44), and noteworthy, the chromosomal location and genetic context of each CRISPR-Cas locus in the genome was exactly the same in all strains, as determined by the analysis of the 10 kb flanking CRISPR loci (data not shown).

The CRISPR repeats are conserved sequences which nucleotide length and structure variable across CRISPR-Cas subtype and bacterial species (Crawley et al., 2018). All the identified loci have a repeat sequence length of 28–29 nucleotides (33.3 and 66% occurrence respectively), with a conserved sequence across strains, with slight variants. The presumed mature CRISPR RNA (crRNA), which comprises a spacer sequence flanked by portions of the CRISPR repeat (Hidalgo-Cantabrana and Barrangou, 2020), was determined using the RNA folding hairpin structure (Figure 5B).

Each CRISPR spacer represents an immunization event providing the bacterium the ability to confront any predator carrying a complementary sequence. The identified CRISPR spacers (*n* = 87) among the 22 *C. acnes* strains, displayed a variable length between 30–33 nucleotides, with 32–33 nt being the most prevalent (66.6 and 31% respectively) (Figure 5C, top panel). A range between 2 and 9 spacers were detected for all strains, with an average of four spacers per strain (Figure 5C, bottom panel). Based on the CRISPR-spacers content, six groups were identified among the 22 strains (Figure 5D, i-vi) when the spacers were aligned and visualized (Nethery and Barrangou, 2019a), with the ancestral spacer on the right, representing a common origin for the strains within each group. The strain HL001 was a unique strain that shared no common ancestral space with any other strain. The representation of the CRISPR repeats allowed to identify the mutated terminal repeat in the strains 09-323, HL082PA2 and HL001PA1 (Figure 5E).

The origin of the invasive nucleic acid corresponding to each spacer of the 22 strains was investigated with BLAST (Camacho et al., 2009). Across the 87 spacers, 47 spacers provided 769 hits with 97–100% identity between the spacer and the protospacer (counterpart of the spacer in the invasive nucleic acid). The *C. acnes* spacers were targeting *Propionibacterium-Cutibacterium* phages and plasmids, with 694 and 75 hits respectively. Interestingly, several strains contained spacers against the same phage, e.g., *Cutibacterium* phage ϕpa28, targeting close regions encompassing the tail protein, minor tail protein and other phage proteins (Figure 5F). The spacer-protospacer alignment and the flanking regions were extracted from CRISPRviz (Nethery and Barrangou, 2019b), to identify the existence of a flanking region carrying the protospacer adjacent motif (PAM) (Horvath et al., 2008; Mojica et al., 2009; Marraffini and Sontheimer, 2010). The PAM motif is a conserved sequence, located at the 5′-end of protospacers in type I CRISPR-Cas systems, which is required for DNA binding and cleavage and is also essential for new spacer acquisition. Ten protospacers, targeting different phages and corresponding to spacers from different strains, were considered for the prediction of the PAM 5′-AA-3′ (Figure 5G). These results, reflecting an AT rich PAM are in concordance with previously PAM sequences described for other type I CRISPR-Cas systems in different genera and species (Mulepati and Bailey, 2013; Leenay et al., 2016; Hidalgo-Cantabrana et al., 2017, 2019).

## Discussion

We have performed comparative genomics analyses in 255 *C. acnes* genomes to elucidate the different genotypes existing in this commensal species related to skin health and disease, understanding the strain diversity and the key genetic features that drive the differentiation among groups. The pan genome analyses displayed an open pan genome which is double the size of the pan genome previously described for a smaller data set of 82 *C. acnes* genomes (Tomida et al., 2013). Although the results of both studies are consistent, previous studies describe a relatively low diversity within this species and an open pan genome. The pan genome representation of gene presence/absence displayed the accessory genes or groups of genes that are responsible for the *C. acnes* clades and sub-clade differentiation. Besides the well-known existence of three clades (Type I-II-III), we have been able to show for the first time the existence of two main sub-clades in clade II (Figure 2). Moreover, this aggrupation was also consistent on the phylogenomics analyses performed based on nucleotide similarity of the core genes, elucidating also several sub-groups for the sub-clade IA (Figure 3), the most prevalent in acnegenic skin.

Over the last decade, different approaches have been used to understand the genetic diversity and phylogenetic groups of *C. acnes* mainly based on single locus sequence analyses (16S rRNA, *RecA*, *tly*) (Scholz et al., 2014; McDowell, 2017; Yang et al., 2019), multilocus sequence typing (MLST) and more recently combined with whole genome sequence analyses (Lomholt and Kilian, 2010; McDowell et al., 2011, 2012, 2013; Bangayan et al., 2020). While some of those typing methods can identify clonal complexes (CC) and sequence types (STs), they cannot differentiate among the sub-clades of type I strains. In fact, the commonly used ribotypes (RTs) in *C. acnes* for strain clustering are based on nucleotide differences among the 16S rRNA sequence, with up to 532 ribotypes (Fitz-Gibbon et al., 2013), while the general clustering classification in clades I, II, III was based on *RecA* sequence, with further classification including the clades and sub-clades IA_1_, IA_2_, IB, IC, II and III, according to MLST (McDowell et al., 2005; Lomholt and Kilian, 2010; Fitz-Gibbon et al., 2013). Moreover, 16S ribotypes and specific SNPs variation has been used to trace *C. acnes* between owner and owner-possessions implementing machine learning models with high accuracy (Yang et al., 2019) but this model does not provide information on the overall strain genotype diversity and taxonomy. Alternatively, Scholz and collaborators defined a SLST typing based on 800 nt sequence specific of *C. acnes* that allows for precise strain classification (Scholz et al., 2014), however, not suitable for understanding the overall genomic content, strain diversity and the key genetic features of each clade and sub-clade.

Such phylogenomic and comparative genomic analyses have been previously used to study commensal and pathogenic bacteria, especially to understand the epidemiology of a rising pathogen and the genetic content that drives pathogenicity (Bhardwaj and Somvanshi, 2017; Laing et al., 2017; Knight et al., 2019; Nethery et al., 2019; Pan et al., 2020). Our results showed how both the pan genome and the phylogenomic analyses with core genes can be used to study *C. acnes* population genetics, understand strain diversity, and differentiate between clades, sub-clades and strains, enabling the identification of diverse genotypes in complex microbiotas with higher robustness than other methods. Moreover, the addition of new genomes to the pipeline will provide comprehensive results, allowing to compare among different studies and strains, while enhancing strain clustering without the need of different typing approaches. Thus, such genome analyses should be used for *C. acnes* typing and taxonomic assignation.

The analyses of the unique genes present in all type I strains allowed to understand the key genetic determinants of this particular clade, highly abundant in acne vulgaris disease. Interestingly, several are related to carbohydrate metabolism and sugar transporters, like glycosyl hydrolase, trehalose transporters *sugA*, PTS transporters and ABC transporters, which can confer an ecological advantage for growth and colonization with a broader metabolic repertoire to scavenge carbohydrates and energy sources (Supplementary Table 1). Trehalose *sugA* is part of the complex transporter system specific for the uptake and catabolism of trehalose typically encompasses a transcriptional regulator, a PTS transporter and a carbohydrate hydrolase (Duong et al., 2006). The presence of both the PTS transporter and the hydrolase enzyme are required for trehalose fermentation, as previously demonstrated in *Lactobacillus acidophilus* with the inactivated-gene mutants unable to grow in the presence of trehalose as carbohydrate source (Duong et al., 2006). Moreover, the ability to uptake this sugar, through translocation of the substrate across the membrane, conferring a metabolic advantage has been described as essential during some stage of infection in *M. tuberculosis* (Kalscheuer et al., 2010). Trehalose is not synthesized by mammalian cells but is produce by a wide range of organisms like bacteria, yeast and fungi (even insects and plants) and can be secreted to the outside of the cell (Ruhal et al., 2013), so the production of trehalose by other members of the skin microbiome could facilitate the overgrowth of type I *C. acnes* strains that has the ability to uptake and catabolize this sugar (trehalose catabolism by type I strains was confirmed in our laboratory, data not shown). Thus, it is important to decipher the interplay within the skin microbiome to understand health and disease in the context of a dynamic and complex microbial community.

Noteworthy, two of the unique genes displayed homology to the type II toxin-antitoxin system HicAB present in *E. coli*, *Salmonella enterica, Citrobacter rodentium*, and *Klebsiella pneumoniae*. HicAB consists of two adjacent genes that constitute an operon, where HicA is the toxin and HicB the antitoxin. HicA toxin activity is based on mRNA interference activity causing cleavage of mRNA, whereas HicB forms a stable complex with HicA to inhibit its function (Daimon et al., 2015; Winter et al., 2018). Toxin-antitoxin systems have been associated with the appearance of persister cells that can act as a reservoir for chronic infections and could presumably have a similar impact in acne proliferation. In this regard, HicA overexpression has been associated with cell growth arrest and an increasing number of persister cells tolerant to several antibiotics, in the opportunistic human pathogen *Burkholderia pseudomallei* and *E. coli* (Butt et al., 2014). In *C. acnes* type I strains, the HicA subunit is a small protein of 64 amino acids (aa), and the HicB subunit consists of 123 aa with a 79.7 and 97% identity to homologs in *E. coli*, respectively. This is relevant since antibiotic treatment has been related to alterations in skin microbial diversity modifying the composition, specifically affecting *C. acnes* and acne severity (Park et al., 2020). The HicAB system could thus constitute an ecological advantage in *C. acnes* type I strains that allows overgrowth under certain physiological conditions of the skin (hormonal changes or drug treatments), which has been described in acne, with a relative increase of type I strains overcoming other *C. acnes* clades and other members of the skin microbiome.

Since the very first *C. acnes* genome sequence was determined almost two decades ago (Bruggemann et al., 2004), several genetic features have been described as potentially implicated in inflammation or tissue damage induction in the host, like co-hemolytic Christie-Atkins-Munch-Peterson (CAMP) factors, hyaluronate lyase (HYL), lipases, and sialidases and endoglycoceramidases, among others, (Achermann et al., 2014; Dreno et al., 2018; McLaughlin et al., 2019). Likewise, cell-surface associated proteins with multiple proline-threonine repeats (PTRs) and inflammatory potential were previously detected.

We here detected the presence of two different genetic variants (alleles) of HYL with differences between a sub-group of type I and type II strains, and no presence in type III strains, in concordance with previously reported results in smaller datasets (Tyner and Patel, 2015; Scholz et al., 2016; Nazipi et al., 2017). These results reflects that the two HYL alleles are a sub-clade characteristic and they do not randomly occur. Moreover, Nazipi and collaborators demonstrated that the two HYL genetic variants has differential activity degrading hyaluronic acid, with the variant present in type IB and type II strains being highly active (dark blue in our Supplementary Figure 1A), and the variant present in type IA (light blue in our Supplementary Figure 1A) barely functional (Nazipi et al., 2017). Thus, the presence of HYL and its presumed effect on the host tissue, destroying certain components of the skin, could promote inflammation in acne vulgaris disease.

This virulent factor PTR-1_PPA0180, uniquely detected in type I strains (Supplementary Figure 1A), also detected within the 51 unique genes occurring in type I strains (Supplementary Table 1), is an ImmA/IrrE family metallo-endopeptidase involved in xenobiotic response, acting as a DNA binding transcriptional regulator under certain environmental conditions. Differential xenobiotic responses (transcriptional profile based on mRNA) of various members of the gut microbiome have been related to the presence of certain drugs, antibiotics as well as pH variations (Maurice et al., 2013). This metallo-endopeptidase has also been identified in other human opportunistic pathogens such as *Mycobacterium tuberculosis, Klebsiella pneumoniae, Staphylococcus aureus*, and *Nocardia farcinica*. Stress response mechanisms, or xenobiotic response to environmental changes, confer tremendous advantages for adaptation to variable environmental conditions, potentially conferring an ecological advantage compared to other species and strains that lack these systems, enabling colonization of and persistence on or in certain environments and human tissues. Other colonization factors like RoxP have been shown to be essential for adherence to and colonization of the skin, but they are ubiquitous in every *C. acnes* strain, and not associate with specific genotypes (Allhorn et al., 2016). This ImmA/IrrE family metallo-endopeptidase endopeptidase exclusively present in type I strains may be considered as a potential indicator of pathogenicity or virulence in *C. acnes* and can also be used as a genetic marker for strain genotyping and taxonomy.

Bacterial porphyrin production, which are pro-inflammatory and related to skin disease, is significantly higher in type I strains, compared to types II and III and also compared to other skin bacteria such as *C. avidum* and *C. granulosum* (Barnard et al., 2020). The absence of the repressor of porphyrin biosynthesis DeoR_PPA0299 has been implicated in higher production of porphyrins in type IA_1_ strains isolated from acnegenic skin (Johnson et al., 2016), and porphyrins production and secretion to the perifollicular area has been related to an inflammatory reaction of the follicle. Thus, our findings on the absence of this repressor in type IA_1_ strains (Supplementary Figure 1A) is consistency with previous literature and correlates with the higher production of porphyrins in the most virulent *C. acnes* genotype, which is predominant in acne vulgaris disease.

The CAMP factors seem widespread among *C. acnes* and not specifically associated with any type. Slight differences in the amino acid identity of the CAMP1_ PPA1340 were detected between type I strains and the type II and type III subsets, ranging from 95.5–100%, with similar findings for CAMP2_PPA0687 (Supplementary Figure 1A). The high distribution of CAMP factors across clades and sub-clades was previously described and transcription regulation may be involved, with CAMP-2 highly expressed in type IA and not in type II strains, according to immunoblotting quantification (Valanne et al., 2005). On the contrary, CAMP-1 is highly produced in type II strains and type IB, but not in type IA which are the majority of *C. acnes* strains.

Overall, previously documented virulent factors are surprisingly widespread among *C. acnes* strains and clades. This contrasts with previous literature reports, though we note differences in amino acid sequences that support consistent *C. acnes* clades clustering in the previously described clades. We report the uniquely occurrence of PTR-1_PPA0180 in type I strains, a genetic marker for this clade, and a potential contributor to pathogenicity. Moreover, we corroborated the existence of different alleles of HYL and the absence of *deoR* in the most virulent sub-clade IA_1_. Besides genomic content, transcriptomic and proteomics analyses of virulence factors should be considered to assess their relative activity across *C. acnes* strains and clades, as previously demonstrated for porphyrins, CAMP factors, dermatan-sulfate adhesins, lipase GehA and others (Valanne et al., 2005; Brzuszkiewicz et al., 2011; Johnson et al., 2016; Barnard et al., 2020).

CRISPR-Cas systems represent the adaptive immune system of bacteria and archaea (Barrangou et al., 2007). Understanding their occurrence and diversity provides insights into the predatory challenges that a bacterium is exposed to within a microbiome (Hidalgo-Cantabrana et al., 2018) while also providing a basis for the development of genome editing tools (Hidalgo-Cantabrana and Barrangou, 2020). We here performed a detailed characterization of CRISPR-Cas systems allowing us to elucidate all the necessary elements to repurpose these systems for downstream applications (Figure 5). Interestingly, CRISPR-Cas systems in *C. acnes* uniquely belong to the type I-E family, and they exclusively occur in type II strains. This association established these CRISPR loci as a specific trait that might be used for strain typing, phylogenetic analysis or taxonomy for clade designation. Moreover, investigating the CRISPR spacers allowed us to elucidate historical and evolutionary relationship among *C. acnes* strains, with six clear groups identified, based on spacers-genotyping (Figure 5D). These results illustrate the potential of CRISPR spacers for genotyping purposes, for identification and traceability of each strain, while reflecting the divergent evolution of *C. acnes* from different ancestors. CRISPR spacers have been used for genotyping of human pathogens previously, providing high-resolution phylogeny for pathogenic bacteria such as *C. difficile* and *Salmonella* (Bachmann et al., 2014; Shariat et al., 2015; Sola et al., 2015; Andersen et al., 2016; Almeida et al., 2017). Noteworthy, the spacer content displayed that the predatory attacks suffered by *C. acnes* are mainly related to phages present in the skin microbiome. Although less efforts have been performed understanding the skin virome, rather than the microbiome, certain metagenomically studies have elucidated that the majority of the skin virome is constituted by bacteriophages against *Propionoibacterium* (*Cutibacterium*), *Staphylococcus*, *Corynebacterium* and *Enterococcus* among other groups (Hannigan et al., 2015, 2017).

Thus, next generation biotherapeutics for skin health could include alternative technologies to modulate the skin microbiome toward a healthy balance to prevent the increase of type I strain population in acne vulgaris, using probiotics (Goodarzi et al., 2020; O’Neill et al., 2020), including specific strains of *C. acnes* (Karoglan et al., 2019) or even skin microbiota transplant (Paetzold et al., 2019). Moreover, naturally existing phages, engineered phages and/or heterologous CRISPR-Cas systems provide new opportunities to alter the skin microbiota composition toward a healthy skin with no impact on other members of the microbiome.

## Conclusion

With diverse *C. acnes* strains coexisting within the skin microbiome, molecular typing methods and whole genome sequencing can provide valuable insights into the roles that different phylogenetic clades may play in health vs. disease. Comparative genomics analyses represent a robust approach enabling large genome data sets comparisons. Our results contribute to the overall understanding of the genetic diversity among the different *C. acnes* clades and sub-clades while elucidating the genetic features that are uniquely present in *C. acnes* type I clade, the most predominant in acne vulgaris disease. For instance, carbohydrate metabolic pathways, transcriptional regulators and stress response mechanisms are key elements that can potentially confer type I strains an ecological advantage for competitive growth and colonization, outcompeting others *C. acnes* clades. Elucidating the differential genetic content among *C. acnes* sub-clades open new avenues to study the mechanisms that drive virulence and pathogenicity, contributing to the development of skin microbiome therapies focused on the selective targeting or manipulation of specific genotypes in acne vulgaris disease.

## Data Availability Statement

The 255 chromosomal genome sequences of the Cutibacterium acnes strains analyzed in this study were those publicly available in the NCBI database as of October 2019 (Supplementary File 1).

## Author Contributions

NC and CH-C designed the study, performed bioinformatic analyses, and participated in the manuscript drafting. AG edited the manuscript. CH-C and RB coordinated and supervised the study and edited the manuscript. All authors approved the final manuscript.

## Conflict of Interest

RB and CH-C are co-inventors on several patents related to CRISPR-Cas systems and their uses. RB is a shareholder of Caribou Biosciences, Intellia Therapeutics, Locus Biosciences, TreeCo, Inari Ag, and CRISPR Biotechnologies. CH-C is a shareholder of Microviable Therapeutics and CRISPR Biotechnologies. AG is an employee of BASF Corporation. The authors declare that this study received funding from BASF Corporation. The funder had the following involvement in the study: edited and approved the final manuscript.

## Publisher’s Note

All claims expressed in this article are solely those of the authors and do not necessarily represent those of their affiliated organizations, or those of the publisher, the editors and the reviewers. Any product that may be evaluated in this article, or claim that may be made by its manufacturer, is not guaranteed or endorsed by the publisher.

## Footnotes

1
https://github.com/sanger-pathogens/Roary/blob/master/bin/create_pan_genome_plots.R

2
http://eggnog5.embl.de/#/app/home

3
https://github.com/sanger-pathogens/Roary/tree/master/contrib/roary_plots

4
https://www.rdocumentation.org/packages/gplots/versions/3.0.3/topics/heatmap.2

5
http://www.microbesonline.org/fasttree/

6
http://tree.bio.ed.ac.uk/software/figtree/

7
https://www.r-graph-gallery.com/heatmap

8
https://github.com/CRISPRlab/CRISPRutils

9
http://nupack.org/

## Supplementary Material

Supplementary Figure 1 Occurrence of virulent genes in *C. acnes*. **(A)** Heatmap of the presence/absence (blue/white) and percentage of identity (blue gradient) of 33 virulent genes (columns) across the 255 *C. acnes* strains used in this study. Hierarchical clustering was performed for both rows and columns and dendrograms were depicted. The main clades of strains were identified, and color coded for type I, type II and type III, with green, blue and red respectively. **(B)** Chromosomal location of the 33 virulent genes displayed in the strain *C. acnes* KPA171202 (subtype IB), with GC-AT content represented as blue-green lines.

Supplementary Figure 2 **(A)** Cas proteins alignment of the type I-E CRISPR-Cas system in *C. acnes* HL110PA4 (as representative) and *E. coli* K12 the displaying the identity (%) at amino acid level. **(B)** Cas proteins alignment of the 45 *C. acnes* strains and *E. coli* K12, and **(C)** corresponding phylogenetic tree of the concatenated Cas proteins.

Supplementary File 1 *Cutibacterium acnes* strains and genome accession numbers.
