# Supplementary material for: Comparative Genomic Analyses and CRISPR-Cas Characterization of Cutibacterium acnes Provide Insights Into Genetic Diversity and Typing Applications
Source: Front Microbiol. 2021 Nov 3;12:758749. doi: 10.3389/fmicb.2021.758749 (PMC8595920; doi:10.3389/fmicb.2021.758749)
Supplement: Supplementary Figure 1 — Occurrence of virulent genes in C. acnes. (A) Heatmap of the presence/absence (blue/white) and percentage of identity (blue gradient) of 33 virulent genes (columns) across the 255 C. acnes strains used in this study. Hierarchical clustering was performed for both rows and columns and dendrograms were depicted. The main clades of strains were identified, and color coded for type I, type II and type III, with green, blue and red respectively. (B) Chromosomal location of the 33 virulent genes displayed in the strain C. acnes KPA171202 (subtype IB), with GC-AT content represented as blue-green lines. [file Presentation_1.zip › Figure S1.PPTX]

## Slide 1
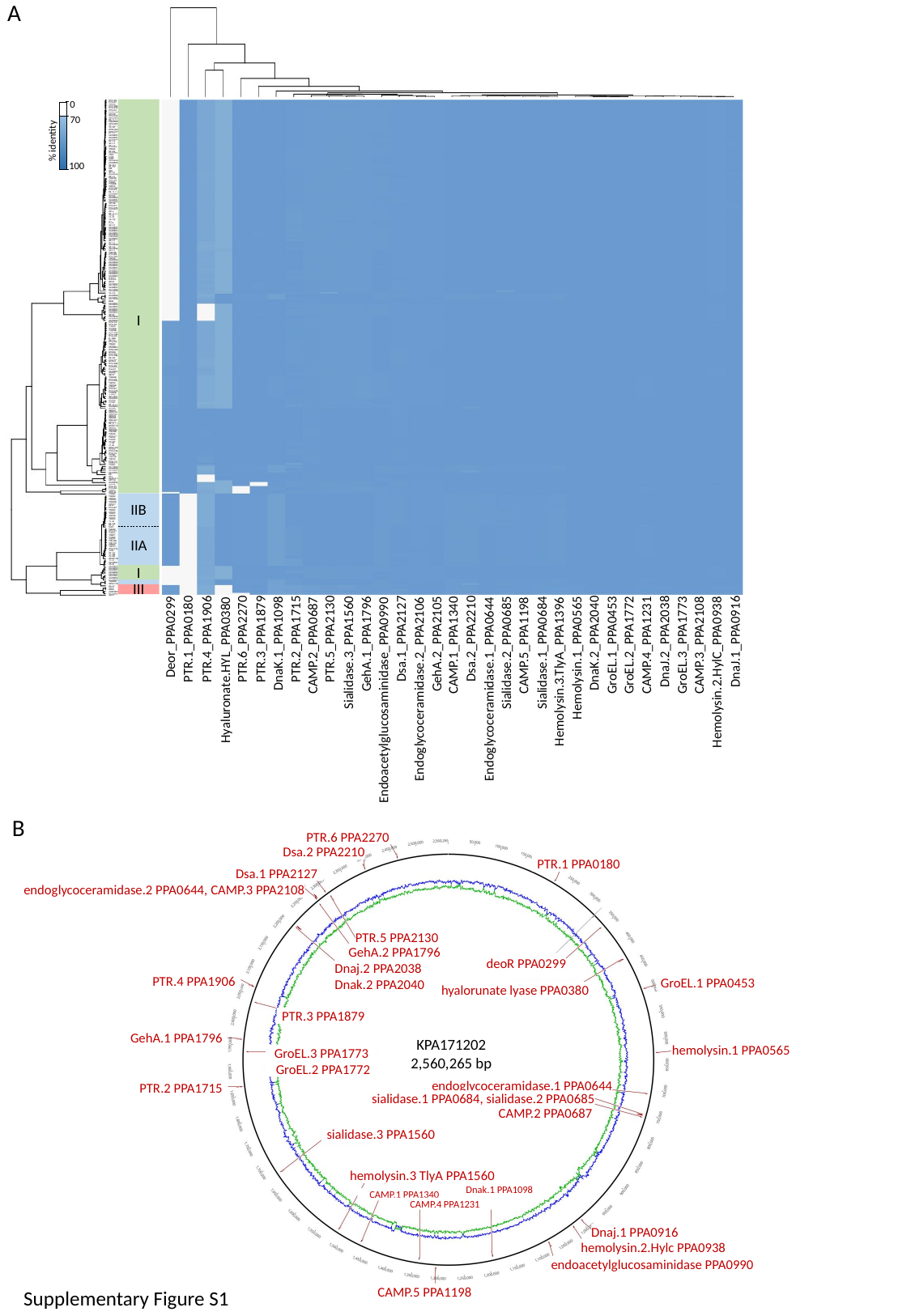

A
0
70
% identity
100
I
IIB
IIA
I
III
Deor_PPA0299
Dsa.1_PPA2127
Dsa.2_PPA2210
PTR.1_PPA0180
PTR.4_PPA1906
PTR.6_PPA2270
PTR.3_PPA1879
PTR.2_PPA1715
PTR.5_PPA2130
DnaJ.2_PPA2038
DnaJ.1_PPA0916
DnaK.1_PPA1098
DnaK.2_PPA2040
GehA.1_PPA1796
GehA.2_PPA2105
GroEL.1_PPA0453
GroEL.2_PPA1772
GroEL.3_PPA1773
CAMP.2_PPA0687
CAMP.1_PPA1340
CAMP.5_PPA1198
CAMP.4_PPA1231
CAMP.3_PPA2108
Sialidase.3_PPA1560
Sialidase.2_PPA0685
Sialidase.1_PPA0684
Hemolysin.1_PPA0565
Hyaluronate.HYL_PPA0380
Hemolysin.3.TlyA_PPA1396
Hemolysin.2.HylC_PPA0938
Endoglycoceramidase.2_PPA2106
Endoglycoceramidase.1_PPA0644
Endoacetylglucosaminidase_PPA0990
B
PTR.6 PPA2270
Dsa.2 PPA2210
PTR.1 PPA0180
Dsa.1 PPA2127
endoglycoceramidase.2 PPA0644, CAMP.3 PPA2108
PTR.5 PPA2130
GehA.2 PPA1796
deoR PPA0299
Dnaj.2 PPA2038
Dnak.2 PPA2040
PTR.4 PPA1906
GroEL.1 PPA0453
hyalorunate lyase PPA0380
PTR.3 PPA1879
KPA171202
2,560,265 bp
GehA.1 PPA1796
hemolysin.1 PPA0565
GroEL.3 PPA1773
 GroEL.2 PPA1772
endoglycoceramidase.1 PPA0644
PTR.2 PPA1715
sialidase.1 PPA0684, sialidase.2 PPA0685
CAMP.2 PPA0687
sialidase.3 PPA1560
hemolysin.3 TlyA PPA1560
Dnak.1 PPA1098
CAMP.1 PPA1340
CAMP.4 PPA1231
Dnaj.1 PPA0916
hemolysin.2.Hylc PPA0938
endoacetylglucosaminidase PPA0990
Supplementary Figure S1
CAMP.5 PPA1198
