# Supplementary material for: Comparative Genomic Analyses and CRISPR-Cas Characterization of Cutibacterium acnes Provide Insights Into Genetic Diversity and Typing Applications
Source: Front Microbiol. 2021 Nov 3;12:758749. doi: 10.3389/fmicb.2021.758749 (PMC8595920; doi:10.3389/fmicb.2021.758749)
Supplement: Supplementary Figure 1 — Occurrence of virulent genes in C. acnes. (A) Heatmap of the presence/absence (blue/white) and percentage of identity (blue gradient) of 33 virulent genes (columns) across the 255 C. acnes strains used in this study. Hierarchical clustering was performed for both rows and columns and dendrograms were depicted. The main clades of strains were identified, and color coded for type I, type II and type III, with green, blue and red respectively. (B) Chromosomal location of the 33 virulent genes displayed in the strain C. acnes KPA171202 (subtype IB), with GC-AT content represented as blue-green lines. [file Presentation_1.zip › Figure S2.PPTX]

## Slide 1
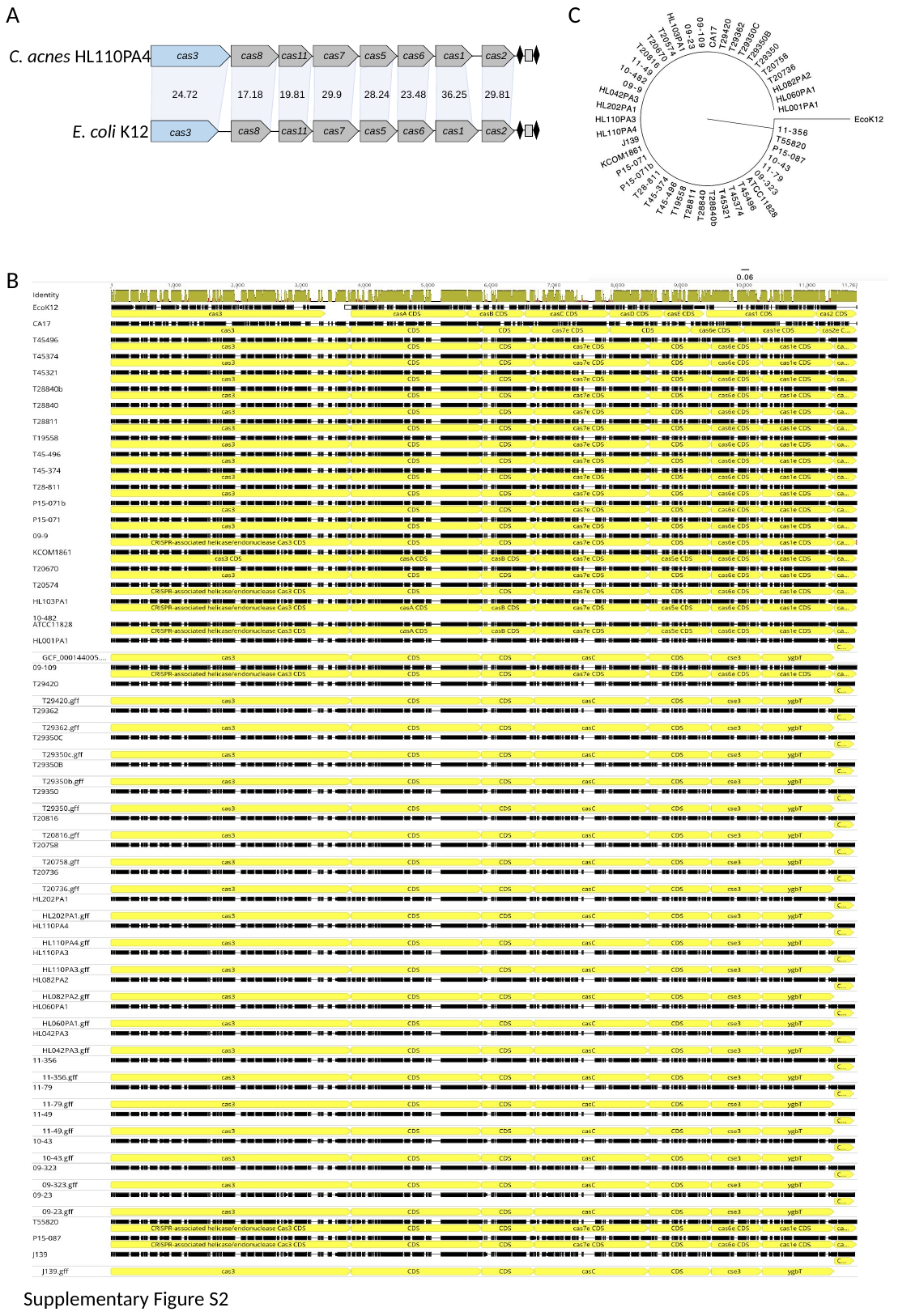

A
C
C. acnes HL110PA4
cas1
cas8
cas11
cas7
cas5
cas6
cas2
cas3
24.72
17.18
19.81
29.9
28.24
23.48
36.25
29.81
E. coli K12
cas11
cas7
cas5
cas6
cas1
cas2
cas8
cas3
B
Supplementary Figure S2
