# Supplementary material for: Comparative Genomic Analyses and CRISPR-Cas Characterization of Cutibacterium acnes Provide Insights Into Genetic Diversity and Typing Applications
Source: Front Microbiol. 2021 Nov 3;12:758749. doi: 10.3389/fmicb.2021.758749 (PMC8595920; doi:10.3389/fmicb.2021.758749)
Supplement: Supplementary Figure 1 — Occurrence of virulent genes in C. acnes. (A) Heatmap of the presence/absence (blue/white) and percentage of identity (blue gradient) of 33 virulent genes (columns) across the 255 C. acnes strains used in this study. Hierarchical clustering was performed for both rows and columns and dendrograms were depicted. The main clades of strains were identified, and color coded for type I, type II and type III, with green, blue and red respectively. (B) Chromosomal location of the 33 virulent genes displayed in the strain C. acnes KPA171202 (subtype IB), with GC-AT content represented as blue-green lines. [file Presentation_1.zip › Table S1.DOCX]

Supplementary Table S1. Fifty-one unique genes detected in all type I strains and not present in type II or type III strains.

| **Gene** | **Prediction** | **Curated annotation** |
| --- | --- | --- |
| GCF_002831725_01457 | *leuC_2*: 3-isopropylmalate dehydratase large subunit | *leuC_2*: 3-isopropylmalate dehydratase large subunit |
| GCF_002831725_01332 | *atpB:* ATP synthase subunit a | *atpB:* ATP synthase subunit a |
| GCF_002831725_01592 | *egsA:* Glycerol-1-phosphate dehydrogenase [NAD(P)+] | *egsA:* Glycerol-1-phosphate dehydrogenase [NAD(P)+] |
| GCF_002831725_01857 | *ndhI:* NAD(P)H-quinone oxidoreductase subunit I | *ndhI:* NAD(P)H-quinone oxidoreductase subunit I |
| GCF_002831725_00629 | *fldH_1:* Phenyllactate dehydrogenase | *fldH_1:* Phenyllactate dehydrogenase |
| GCF_002831725_00628 | *pth:* Peptidyl-tRNA hydrolase | *pth:* Peptidyl-tRNA hydrolase |
| GCF_002831725_00934 | *rsfS*: Ribosomal silencing factor RsfS | *rsfS*: Ribosomal silencing factor RsfS |
| GCF_002831725_01267 | *aroE*: Shikimate dehydrogenase NADP+ | *aroE*: Shikimate dehydrogenase NADP+ |
| GCF_002831725_01269 | *aroK*: Shikimate kinase | *aroK*: Shikimate kinase |
| GCF_002831725_01925 | Trehalose transport system permease protein SugA | Trehalose transport system permease protein SugA |
| GCF_002831725_02208 | Hypothetical protein | AAA family ATPase |
| GCF_002831725_02260 | Hypothetical protein | ABC transporter permease |
| GCF_002831725_01471 | Hypothetical protein | Acetolactate synthase |
| GCF_002831725_01836 | Hypothetical protein | Actinobacterial surface-anchored domain protein |
| GCF_002831725_01334 | Hypothetical protein | Cyanate permease |
| GCF_002831725_00630 | Hypothetical protein | D-lactate dehydrogenase |
| GCF_002831725_00251 | Hypothetical protein | Fadr transcriptional regulator |
| GCF_002831725_00333 | Hypothetical protein | AAA ATPase |
| GCF_002831725_01213 | Hypothetical protein | Anthranilate synthase component I |
| GCF_002831725_02132 | Hypothetical protein | Glycosyl hydrolase |
| GCF_002831725_00960 | Hypothetical protein | HTH-type transcriptional regulator YdcR |
| GCF_002831725_00267 | Hypothetical protein | ImmA/IrrE family metallo-endopeptidase |
| GCF_002831725_00109 | Hypothetical protein | Metal-dependent amidase/aminoacylase/carboxypeptidase |
| GCF_002831725_00181 | Hypothetical protein | N-acetyltransferase |
| GCF_002831725_01644 | Hypothetical protein | N-acetyltransferase |
| GCF_002831725_00323 | Hypothetical protein | Phage holin |
| GCF_002831725_01613 | Hypothetical protein | Predicted extracellular nuclease |
| GCF_002831725_01591 | Hypothetical protein | PTS sugar transporter |
| GCF_002831725_00133 | Hypothetical protein | Sigma70 family RNA pol. |
| GCF_002831725_00751 | Hypothetical protein | Toxin-antitoxin system HicA |
| GCF_002831725_00752 | Hypothetical protein | Toxin-antitoxin system HicB |
| GCF_002831725_01274 | Hypothetical protein | Transcription antitermination factor NusB |
| GCF_002831725_01612 | Hypothetical protein | Transcriptional regulator |
| GCF_002831725_00767 | Hypothetical protein | YdcF (CDD 99750) |
| GCF_002831725_00787 | Hypothetical protein | Hypothetical protein |
| GCF_002831725_00561 | Hypothetical protein | Hypothetical protein |
| GCF_002831725_02264 | Hypothetical protein | Hypothetical protein |
| GCF_002831725_00385 | Hypothetical protein | Hypothetical protein |
| GCF_002831725_01107 | Hypothetical protein | Hypothetical protein |
| GCF_002831725_01967 | Hypothetical protein | Hypothetical protein |
| GCF_002831725_00068 | Hypothetical protein | Hypothetical protein |
| GCF_002831725_00768 | Hypothetical protein | Hypothetical protein |
| GCF_002831725_01033 | Hypothetical protein | Hypothetical protein |
| GCF_002831725_01488 | Hypothetical protein | Hypothetical protein |
| GCF_002831725_01489 | Hypothetical protein | Hypothetical protein |
| GCF_002831725_01688 | Hypothetical protein | Hypothetical protein |
| GCF_002831725_01698 | Hypothetical protein | Hypothetical protein |
| GCF_002831725_01969 | Hypothetical protein | Hypothetical protein |
| GCF_002831725_00358 | Hypothetical protein | Hypothetical protein |
| GCF_002831725_00478 | Hypothetical protein | Hypothetical protein |
| GCF_002831725_01821 | Hypothetical protein | Hypothetical protein |

* GCF_002831725_xx represents the gene locus_tag identifier based on *C. acnes* 09-29 genome (GCF_002831725)
