# Supplementary material for: Comparative Genomic Analyses and CRISPR-Cas Characterization of Cutibacterium acnes Provide Insights Into Genetic Diversity and Typing Applications
Source: Front Microbiol. 2021 Nov 3;12:758749. doi: 10.3389/fmicb.2021.758749 (PMC8595920; doi:10.3389/fmicb.2021.758749)
Supplement: Supplementary Figure 1 — Occurrence of virulent genes in C. acnes. (A) Heatmap of the presence/absence (blue/white) and percentage of identity (blue gradient) of 33 virulent genes (columns) across the 255 C. acnes strains used in this study. Hierarchical clustering was performed for both rows and columns and dendrograms were depicted. The main clades of strains were identified, and color coded for type I, type II and type III, with green, blue and red respectively. (B) Chromosomal location of the 33 virulent genes displayed in the strain C. acnes KPA171202 (subtype IB), with GC-AT content represented as blue-green lines. [file Presentation_1.zip › Table S2.DOCX]

Supplementary Table S2. Unique genes detected in sub-clade IIA and sub-clade IIB.

| **Gene** | **Prediction** | **Curated annotation** |
| --- | --- | --- |
| **Unique genes in IIA** |  |  |
| GCF_005937155_02242 | *gatC2*: PTS system galactitol-specific EIIC component | gatC2: PTS system galactitol-specific EIIC component |
| GCF_005937155_02246 | Hypothetical protein | NAD-dependent glycerol-3-phosphate dehydrogenase |
| **Unique genes in IIB** |  |  |
| GCF_000144465_00966 | *ctaA*: Heme A synthase | *ctaA*: Heme A synthase |
| GCF_000144465_00971 | *malG*: Maltose transport system permease protein | *malG*: Maltose transport system permease protein |
| GCF_000144465_00972 | *malF*: Maltose transport system permease protein | *malF*: Maltose transport system permease protein |
| GCF_000144465_00973 | *malE*: Maltose-binding periplasmic protein | *malE*: Maltose-binding periplasmic protein |
| GCF_000144465_00918 | Hypothetical protein | MFS transporter, major facilitator family protein |
| GCF_000144465_01103 | *DprA*: DNA processing, | *DprA*: DNA-protecting protein |
| GCF_000144465_01100 | Hypothetical protein | Hypothetical protein |
| GCF_000144465_01212 | Hypothetical protein | ABC transporter permease |
| GCF_000144465_01845 | Hypothetical protein | Dinitrogenase iron-molybdenum cofactor |

* GCF_005937155_xx represents the gene locus_tag identifier based on *C. acnes* T45496 genome (GCF_005937155)

** GCF_000144465_xx represents the gene locus_tag identifier based on *C. acnes* HL050PA2 genome (GCF_000144465)
