# Supplementary material for: Comparative Genomic Analyses and CRISPR-Cas Characterization of Cutibacterium acnes Provide Insights Into Genetic Diversity and Typing Applications
Source: Front Microbiol. 2021 Nov 3;12:758749. doi: 10.3389/fmicb.2021.758749 (PMC8595920; doi:10.3389/fmicb.2021.758749)
Supplement: Supplementary Figure 1 — Occurrence of virulent genes in C. acnes. (A) Heatmap of the presence/absence (blue/white) and percentage of identity (blue gradient) of 33 virulent genes (columns) across the 255 C. acnes strains used in this study. Hierarchical clustering was performed for both rows and columns and dendrograms were depicted. The main clades of strains were identified, and color coded for type I, type II and type III, with green, blue and red respectively. (B) Chromosomal location of the 33 virulent genes displayed in the strain C. acnes KPA171202 (subtype IB), with GC-AT content represented as blue-green lines. [file Presentation_1.zip › Table S3.DOCX]

Supplementary Table S3. Thirty-three virulent factors used as query for the analyses

| **Gen-ORF** | **Information** | **Effect on the host** |
| --- | --- | --- |
| CAMP-1_PPA1340 | CAMP factor | Hemolytic, cytotoxic, inflammation, colonization |
| CAMP-2_PPA0687 | CAMP factor | Hemolytic, cytotoxic, inflammation, colonization |
| CAMP-3_PPA2108 | CAMP factor | Hemolytic, cytotoxic, inflammation, colonization |
| CAMP-4_PPA1231 | CAMP factor | Hemolytic, cytotoxic, inflammation, colonization |
| CAMP-5_PPA1198 | CAMP factor | Hemolytic, cytotoxic, inflammation, colonization |
| DeoR_PPA0299 | Repressor gene of porphyrin synthesis | - |
| DnaJ-1_PPA0916 | Heat shock protein | Inflammation |
| DnaJ-2_PPA2038 | Heat shock protein | Inflammation |
| DnaK-1_PPA1098 | Heat shock protein | Inflammation |
| DnaK-2_PPA2040 | Heat shock protein | Inflammation |
| DsA-1_PPA2127 | Dermatan sulphate adhesin | Colonization, adhesion, inflammation |
| DsA-2_PPA2210 | Dermatan sulphate adhesin | Colonization, adhesion, inflammation |
| Endo-β-N-acetylglucosaminidase_PPA0990 | oligosaccharides processing | Tissue damage |
| Endoglycoceramidase-1_PPA0644 | Catalyzes β-glycosidic linkage between oligosaccharides and ceramides | Tissue damage |
| Endoglycoceramidase-2_PPA2106 | Catalyzes β-glycosidic linkage between oligosaccharides and ceramides | Tissue damage |
| GehA-1_PPA1796 | Triacylglycerol lipase | Tissue damage, inflammation |
| GehA-2_PPA2105 | Triacylglycerol lipase | Tissue damage, inflammation |
| GroEL-1_PPA0453 | Heat shock protein | Inflammation |
| GroEL-2_PPA1772 | Heat shock protein | Inflammation |
| GroEL-3_PPA1773 | Heat shock protein | Inflammation |
| Hemolysin-1_PPA0565 | Hemolysin | Hemolytic |
| Hemolysin-2(HylC)_PPA0938 | Hemolysin | Hemolytic |
| Hemlosyin-3(Tly-A)_PPA1396-PPARS07050 | Hemolysin | Hemolytic |
| Hyaluronate-HYL_PPA0380 | Hyaluronate Lyase | Tissue damage |
| PTR-1_PPA0180 | Proline-Threonine repeat pattern | Colonization, adhesion, inflammation |
| PTR-2_PPA1715 | Proline-Threonine repeat pattern | Colonization, adhesion, inflammation |
| PTR-3_PPA1879 | Proline-Threonine repeat pattern | Colonization, adhesion, inflammation |
| PTR-4_PPA1906 | Proline-Threonine repeat pattern | Colonization, adhesion, inflammation |
| PTR-5_PPA2130 | Proline-Threonine repeat pattern | Colonization, adhesion, inflammation |
| PTR-6_PPA2270 | Proline-Threonine repeat pattern | Colonization, adhesion, inflammation |
| Sialidase-1_PPA0684 | Cleave sialoglycoconjugates | Tissue damage |
| Sialidase-2_PPA0685 | Cleave sialoglycoconjugates | Tissue damage |
| Sialidase-3_PPA1560 | Cleave sialoglycoconjugates | Tissue damage |

* Locus code (PPA_) is based on *C. acnes* KPA171202
